# Diverging roles of domain-specific anxieties in number-space associations. Insights from a multi-directional number line paradigm

**DOI:** 10.1007/s00426-025-02179-0

**Published:** 2025-09-30

**Authors:** Sophie J. Leonard, Flavia H. Santos

**Affiliations:** 1 School of Psychology, University College Dublin, Dublin, Ireland; 2 Institute of Education, University College London, London, United Kingdom

**Keywords:** Math anxiety, Spatial anxiety, Numerical cognition, Spatial cognition, Spatial-numerical associations

## Abstract

Rapidly evolving scientific, technological and societal advancements depend on those who are accomplished in mathematics, so uncovering the underpinnings of maths performance remains a valuable topic of research. The interplay between numerical and spatial aspects of cognition is of interest in this respect, and interference in this relationship from domain-specific anxieties present potential long-term consequences for overall maths learning and achievement. This study examines the impacts of maths anxiety and spatial anxiety on visuo-spatial elements of mathematical performance i.e. number line estimation. Across three separate experiments, we examine how different variations of the number line estimation task (i.e. directionality, range and unbounding) tap into spatial aspects of numerical cognitive interaction with domain-specific anxieties. Secondarily, we explore the pertinent relationship between these emotional factors and gender. We implemented three adaptations of the multi-directional number line task to simultaneously assess elements of individual differences in spatial-numerical associations. The paradigm was administered online to 471 participants in the United Kingdom across three studies. Regression models indicated that while directional number line estimation was predicted by maths anxiety, no relationship was found for spatial anxiety in any of the three experiments. While no differences in gender were found in task performance, significant differences existed in both anxiety domains. We discuss results in the context of the solid, yet elusive relationships between numerical and spatial processing and the affective underpinnings of mathematics performance in adults.

**Supplementary Information:**

The online version contains supplementary material available at 10.1007/s00426-025-02179-0.

## Introduction

It is often theorised that an ability to relate and organise numerical and spatial processing can benefit mathematical ability (Hawes & Ansari, 2020). Yet, Cipora and colleagues (2018) raise the point that in many circumstance spatial-numerical associations (SNAs) may not be as evidently linked to mathematics ability and in actuality, relatively little is still known about how and why spatial processes relate to numerical facets of thinking (Atit et al., 2021). Constant and imminent scientific advances yield an ever-growing demand for those with skills in fields of science, technology, engineering and maths (STEM), success in which is predicted by both mathematical and spatial ability (Wai et al., 2009). Still, emotional components of both maths and spatial tasks remain marked inhibitors of performance and attitudes of students throughout education and into adulthood (Akin & Kurbanoglu, 2011; Carey et al., 2016). Furthermore, such emotional factors present strong associations with gender, which also contribute to perceived discrepancies in performance (Devine et al., 2012; McCoy et al., 2021). In light of this, it is particularly important to understand the role of domain-specific anxieties in inhibiting STEM development (Atit et al., 2021; Trassi et al., 2022). In this study, we look specifically at how maths anxiety and spatial anxiety may alter the number-space link using a novel design to scrutinise cognitive facets of spatial-numerical performance.

## Number-space research and the ‘internal number line’

Grasping various numerical concepts such as number sense, magnitude processing and ordinality rely on an inherent understanding of space and distance (Bobis 2008; Cipora et al., 2020; Gilligan et al., 2019; Lyons et al., 2014). The most prominent consistent empirical observation to characterise such a process, is that of the spatial-numerical association of response codes (SNARC); a certain spatial organisation of numbers in the brain or the ‘Mental Number Line’, whereby smaller numbers are associated with the left side of space, while larger numbers are associated with the right, particularly among those with left-to-right reading and writing habits, in which many linguistic and numerical systems are learnt and written in left-to-right format (Dehaene et al., 1993, 2003; Zorzi et al., 2012). This is evident throughout the behavioural literature, with evidence suggesting the SNARC effect is often observed to be replicated but reversed in cultures such as Arabic, corresponding with the right-to-left writing direction (Shaki et al., 2012; Zebian, 2005). The SNARC effect specifically describes the phenomenon where people respond faster with the left hand to smaller numbers and with the right hand to larger numbers. Evidence of the SNARC effect has also been found in new-born babies; young children; and even non-human animals suggesting an innate spatial bias in numerical processing (Cooney et al., 2021; DiGiorgio et al., 2019; Prete & Tomassi, 2020; Rugani et al., 2015).

Observations in research implementing the SNARC effect, highlight a connection between number magnitude processing and spatial processing in response tasks, though this amasses to just one facet of Spatial Numerical Associations (SNAs), a broader term which refers to the general tendency to associate smaller numbers with the left side and larger numbers with the right side in space (For a review, see Toomarian and Hubbard, 2018). It should be noted that SNAs have been observed to occur across multiple orientations and internal axes in both children and adults depending on multiple factors such as individual difference and task context as described by Cipora and colleagues (2018) in their chapter on relations between space and numbers (See also Cooney et al., 2021; Shaki & Fischer, 2018; Wiemers et al., 2017, for evidence of varying representations of SNAs).

Another means of observing SNAs is with a number line estimation task, requiring participants to place a target number along a finite line in accordance with a specified range of numbers (Berteletti et al., 2015; Siegler & Booth, 2005). While number line estimation is used in research as a means of directly tapping into spatial facets of numeracy, individual differences in this cognitive process are apparent depending on several factors. For example, the direction, or indeed the salience, of an individual’s internal number line may differ depending on circumstances like their educational or cultural background or their mathematics expertise (Cipora et al. 2018; See Gobel et al., 2011, for a review on cultural variations). It is also postulated that presenting number line estimations with numerical ranges of varying complexities may be driven by different spatial strategies as well as increasing cognitive load and demand on working memory (Hurst & Hurrell, 2014; Nunez-Pena et al., 2019). Furthermore, variations in the number line estimation paradigm itself can also impact how the task isolates more spatial processes associated with numeracy. Cohen and Blanc-Goldhammer (2011) devised an “unbounded number-line estimation” task, in which end-points are removed from the number line, and only a starting point and unit of distance along the line is displayed instead. It is argued that this paradigm may present a more refined means of studying estimation strategies and isolating spatial facets of numerical processing (Link, Nuerk and Moeller, 2014; Reinert and Moeller, 2021). Link and colleagues (2014) argue that bounded and unbounded number lines may actually tap into different aspects of spatial numerical processing with performance on the traditional bounded number line being supported by more numerical competencies such as proportion judgment and arithmetic (Barth & Paladino, 2011; Link, Nuerk and Moeller, 2014).

While most research has focused on horizontal number line estimation, emerging evidence suggests vertical and reversed horizontal SNAs may be equally or even more salient, particularly when typical directional expectations are disrupted (Di Lonardo et al., 2020; Leonard et al., 2023; Shaki & Fischer, 2018). Simms et al. (2013) also found that children perform comparably on both horizontal and vertical number line estimation tasks, supporting the idea that spatial-numerical mappings are not strictly bound to a single axis. Presenting number lines in multiple orientations may allow for assessment of the robustness and variability of spatial-numerical representations in different configurations, providing insight into the flexibility of the internal number line.

From the above, we infer that innate, individual and educational factors shape this intrinsic number line (Fischer et al., 2009; Hesse and Bremmer, 2017). The association between domain-specific anxieties and spatial-numerical associations using both cognitive and neurophysiological measures is of interest to researchers in determining the underpinnings of the number-space links and the manner in which they might impact performance on mathematical (and indeed more broadly spatial) tasks (Beilock & Maloney, 2015; Georges et al., 2016; Núñez-Peña et al., 2021; Viarouge et al., 2014). In light of this literature, it seems important to consider the above elements when examining the role of individual difference in the form of emotional facets on spatial numerical associations on the internal number line.

### Mathematics anxiety in number-space research

The section above suggests that SNAs may be impacted by elements of individual difference, and one such element which presents a challenge for researchers and educators is the emotional construct of Mathematics Anxiety (Chang & Beilock, 2016; McCoy et al., 2021). Mathematics Anxiety (MA) is a combination of negative thoughts, feelings, physiological responses and avoidance behaviours, which presents itself in some individuals while dealing with mathematical tasks or concepts, and is ultimately destructive for both accuracy and reaction time during mathematics performance (Ashkraft & Krause, 2007; Richardson & Suinn, 1972). It is considered to be related to, but a separate construct from, test anxiety, general anxiety or state anxiety (Barroso et al., 2021; Carey et al., 2017; Hembree, 1990; Stöber & Pekrun, 2004); Suárez-Pelliccioni et al., 2015). MA is observed in populations of both men and women worldwide as early as first grade (Barroso et al., 2021). Though MA can seemingly occur independently from mathematical ability, its presence can be associated with lower numerical capabilities and often hampers maths learning mastery, maths grades and overall motivation to do well in maths over time regardless of baseline numerical capacity (Ashkraft and Krause, 2007; Carey et al., 2017; Cohen & Rubinstein, 2021; Daker et al., 2022). An established line of thought stemming from such findings states that MA is not necessarily a result of a numerical learning difficulty, rather affective ruminations and worry which deplete cognitive resources associated with typical mathematical processing, serving to hinder mathematical learning and performance (Maloney et al., 2012). The cycle continues, with inhibitions in mathematical learning due to avoidance which occur due to MA further bolstering the emotional deficit (Carey et al., 2016).

In light of its potentially detrimental consequences for educational achievement, career choices and subsequent quality of life, establishing the underpinnings of MA and how it may impact specific cognitive elements of numerical processing present a topic of interest (Ashkenazi & Cohen, 2021; Ritchie & Bates, 2013). The negative associations between MA and maths performance results in different brain activations between typically developing children and those with a mathematical learning disorder, with the educational and emotional consequences of this extending into adulthood (Hartwright et al., 2018; Kucian et al., 2018; Young et al., 2012).

Research on the theorised cognitive impacts of MA has found it is indicative of trouble processing the verbal stimuli associated with arithmetic, perhaps due to increased cognitive resources needed to hold a verbal calculation in working memory, while simultaneously attempting to solve the problem and manage symptoms of MA (Mammarella et al., 2015; Passolunghi et al., 2020). It is found to be not only predictive of accuracy and response times on purely arithmetic tasks, but also on visuospatial number line estimation tasks and even more ‘traditional’ spatial abilities such as mental rotation or the gauging of spatial relationships (Blajenkova et al., 2006; Maloney et al., 2012; Passolunghi et al., 2020). While maths anxious individuals tend to be both less accurate and slower in both calculation and number lines estimation than those with lower MA, such outcomes can differ depending on a number of factors (Sokolowski et al., 2019). Participants with a degree of educational experience in mathematics may not necessarily differ in accuracy on more basic calculations as these numerical representations may become automatised over time, leaving response time as a sole predictor of anxiety related difference (Ashcraft & Moore, 2009). While on tests of “non-familiar number lines”, those with higher levels of MA may differ in accuracy of estimation but not necessarily in response time to their low MA peers (Núñez-Peña et al., 2019). For such reasons, the present study will investigate both measures when examining the relationship of MA to numerical and spatial tasks.

The general trend which exists in the literature of higher reported MA among women could be, at least partially, explained by potential differences in processing visuospatial imagery in addition to societal factors or a greater willingness among women to report on emotional experience as previously hypothesised (Ashcraft & Moore, 2009; Maloney et al., 2012; Núñez-Peña et al., 2019; Paul et al., 2022). As such, if research on MA has yielded evidence of association with the spatial facets of cognitive processing, then it is rational to assume specific emotional components of spatial thinking may also play a role in the spatial underpinnings of numerical processing (Georges et al., 2015; Lauer et al., 2018).

## Spatial anxiety in number-space research

Spatial Anxiety (SA) has been coined as a domain-specific and multi-faceted form of anxiety which arises when faced with tasks such as way-finding, mental rotation and manipulation or even perspective-taking (Alvarez-Vargas, 2020). While the underpinnings of SA remain largely unexplored relative to other forms of anxiety, it presents similar symptoms and trajectories to MA, emerging early in education with largely negative consequences for spatial performance over time (Malanchini et al., 2017). Again, similar to the case of MA, since spatial reasoning places a high demand on both visual-spatial and verbal working memory resources, this could also explain the presence of SA when solving rotational or navigational tasks (Alvarez-Vargas, 2020; Gunderson et al., 2012).

Although there is sufficient grounds to consider SA a separate construct from either MA or other forms of anxiety, consisting of its own genetic and environmental influences, SA and MA have been found to correlate moderately (Ferguson et al., 2015; Malanchini et al., 2017). SA has been found to predict performance on both large and small scale spatial tasks, i.e., wayfinding and mental manipulation, respectively (Geer et al., 2019; Sokolowski et al., 2019). In fact, both SA and MA are reported as predictors of task accuracy, with highly maths anxious as well as spatially anxious individuals being more likely to report anxiety in relation to wayfinding (Ferguson et al., 2015). This presents a curious point, since large scale spatial skills (e.g. navigation or wayfinding) and small scale spatial skills (e.g. mental rotation and manipulation of objects) are often considered cognitively separate constructs; with the expectation that anxiety relating to small scale spatial processes may be more predictive of spatial-numerical associations in mathematics (Hegarty & Waller, 2004; Malanchini et al., 2017; Wang et al., 2014, 2020). An interesting addition is that SA also seems to play a role in basic numerical performance, with developmental research finding scores on the child spatial anxiety scale, incorporating both large and small scale spatial questions, moderate the longitudinal relationship between spatial perception and subitizing (Ouyang et al., 2022). It remains to be seen how this aspect of SA may relate to spatial numerical associations.

Similar to the literature on MA, gender differences are also apparent in this construct, with women consistently showing higher levels of SA than men, which in turn can result in negative consequences for spatial performance (Alvarez-Vargas et al., 2020; Sokolowski et a., 2019). Sokolowski and colleagues (2019) demonstrated that gender differences in MA were actually mediated by spatial factors rather than numerical factors; with SA acting as an even larger mediator of this gender difference than spatial ability. This finding presents the possibility that the gender discrepancies in MA seen consistently in numerical research, could be underpinned by an avoidance of the spatial processes involved in mathematical tasks rather than purely numerical processes. Further explorations of the overlap between these measures of domain specific anxiety in their prediction on numerical and spatial processing may function to resolve some discord.

## The present study

Despite an array of research exploring spatial-numerical associations and how they may link to emotional components of mathematics, questions still stand regarding the extent and means by which these emotional predictors converge or diverge within number-space spatial processing. Furthermore, disagreement is evident in the literature regarding the rigidity and direction (if any) of the internal mental number line in different individuals under different conditions, and to what extent this visuo-spatial deviation is influenced by emotional factors (Hartwright et al., 2018; Maloney & Beilock, 2012). The present study utilises a multi-directional number line task, to assess how MA and SA may work to predict both purely numerical elements of maths performance as well as those with a very clear visuospatial element on varying types of number lines. While the current paradigm has been used in educational assessments with school children, it has not yet been tested with adults or to explore relations between SNAs and emotional factors (Leonard et al., 2023).

We conducted three experiments with university students. In the first experiment, we employ a fast paced bounded number line estimation task examined from multiple directions in addition to arithmetic controls to uncover how number-space processes as well as spatial and mathematical anxieties converge.

To further isolate the spatial contributions in number-line estimation, we incorporated two specific task manipulations: an increased numerical range (0-1000) and an unbounded number line. While a wider range may superficially appear more complex, it is also possible that these larger, less frequently encountered number ranges are less automatised than a standard 0-100 range, particularly for adults (Di Lonardo et al., 2020; Siegler & Opfer, 2003). The unbounded version, removes a visual endpoint and requires estimations based on internalised units, offering a purportedly purer measure of spatial-numerical mapping (Cohen & Blanc-Goldhammer, 2011; Link et al., 2014; Reinert & Moeller, 2021). Including both variations enables us to probe whether different paradigms tap distinct spatial estimation strategies, and how these are modulated by individual difference variables such as MA and SA. We account for potential impacts that variations in complexity and “boundedness” of the number line estimation paradigm may have on spatial processing during this task. In accordance, two further versions of the task are employed whereby we incorporate an increased number range (*Experiment 2*) and an unbounded number line (*Experiment 3*) in the cognitive task to examine whether they may exhibit more salient interactions with MA and SA than the original task due to perceived differences in the underlying spatial processes involved with both.

Due to the societal discourse surrounding gender discrepancies in numerical and spatial research, we will secondarily explore the role of gender in potential associations between these domain-specific anxieties and task performance. As such, the following hypotheses will be implemented and examined using multiple regression analysis to answer our research questions in the current study:

- Hypothesis 1: *(Experiment 1)* Due to the close nature of MA and SA in the literature, and previous observations of MA as an inhibitor in both arithmetic and spatial aspects of mathematics performance, we propose that higher levels of MA and SA will yield significant negative relationships with performance (accuracy and reaction time) on number line trials.
- Hypothesis 2: *(Experiment 2)* Higher levels of MA and SA will negatively affect performance (accuracy and reaction time) on number line trials, due to the increased number range and complexity of the number line task.
- Hypothesis 3: *(Experiment 3)* Higher levels of MA and SA will negatively affect performance (accuracy and reaction time) on number line trials due to the unbounded number line again presented across 4 different directional orientations, which is perceived to be a more refined measure of spatial processing in number line estimation.

Additional exploratory hypothesis: We expect gender to play a significant role in any potential associations between levels of MA or SA and task performance across all experiments.

## Experiment 1

### Methods

#### Participants

Informed consent was obtained from all participants prior to commencement in accordance with the Declaration of Helsinki and the study received ethical approval from the University’s Human Research Ethics Committee. A sample size determination analysis for a within-subjects design using G*Power (Faul et al., 2007) was conducted for a multivariate analysis using Cohen’s f² = 0.15, accounting for the 4 dependent variables and predictor variables of MA and SA yielded a minimum sample of 129 participants. The participants included in data analysis were 193 adults recruited online (Mean age = 29.5, SD = 10.52) using advertisements targeting residents of the United Kingdom who had received their primary mathematics education through English. The percentages of women and men were 63.3% versus 32.2%. The percentage of participants who reported having a career or expertise in Science Technology Engineering and Math (STEM) was 39.0%, while 64.2% of participants reported having completed third level education. Right handed participants made up 86.3% of the sample and 92.6% of participants reported using their right hand to control their laptop/computer cursor. No participants reported a diagnosis of dyscalculia or a mathematics learning disorder.

## Materials and measures

### Cognitive task

**Fig. 1:**
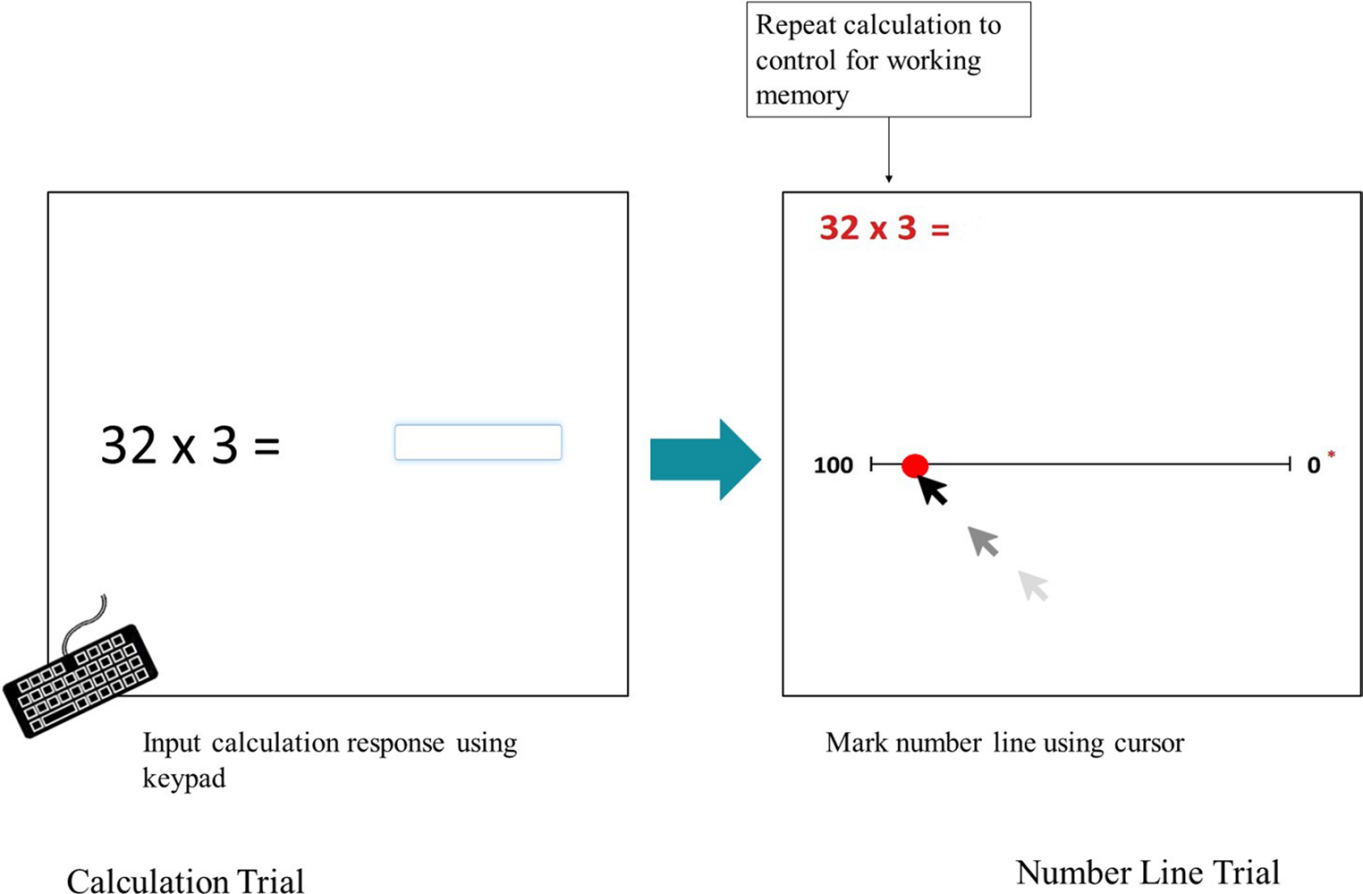
Trial schema depicting one number line trial. Participants received a break half-way through the task at the 48 trial mark

The multi-directional number line task was designed to assess visuo-spatial numerical abilities in their impact on anxiety. The task was both created and administered using Gorilla Experiment Builder (Anwyl-Irvine et al., 2020) (https://gorilla.sc/*)* and was composed of 96 trials encompassing the following conditions: number line trial direction (Left-right, Right-left, Bottom-top, Top-bottom). Each number line trial is preceded by one arithmetic calculation to control for general arithmetic ability against number line estimation. Each calculation control was presented in horizontal format with one operator only. This task was designed such that each trial was preceded by a symbolic arithmetic calculation involving the same numerical value. Practically, this approach ensures that participants engage with numerically meaningful values and allow for control of arithmetic accuracy. Theoretically, this design was informed by prior research suggesting that arithmetic and spatial-numerical reasoning recruit overlapping cognitive and neural processes (e.g., Montefinese & Semenza, 2018). By presenting the same mathematical content in both symbolic and spatial formats, we aimed to investigate whether performance and anxiety responses varied across these representational modalities, providing insight into the extent to which spatial and arithmetic processing share underlying mechanisms. Answers ranged between 0 and 100 and each calculation corresponded with the target number for the number line trial to follow. See Fig. 1 for a graphic of the trial mechanism. Calculation controls were balanced to include the four operators (addition, subtraction, multiplication, division), to include equal numbers of responses higher and lower than the midpoint of ‘50’, and both one and two digits calculations were used. In this experiment, each condition was counterbalanced across the 96 trials and presented in randomised order. All stimuli were presented in black font size 48 in the centre of a white background. In the calculation trials, participants used their keyboards to input their response in the box provided on screen and pressed the ‘Spacebar’ key to submit. The same target was then presented in the top left hand corner on the number line screen (to reduce working memory load), with the number line in the centre of the screen.

Number line stimuli in black ink ranged from 0 to 100 and the stimulus line measured 1200 pixels in both horizontal and vertical trials. A red asterisk was placed by the zero in each trial to draw participant attention to the number line direction. In the current task, the number line orientations were presented randomly, therefore orientations were inter-mixed and any one orientation could precede or follow another. Since the task was administered online, screen resolution and participant distance from screen could not be controlled, though the task design ensured it was only run on a laptop or PC and the task was programmed to fit the display of each screen, so proportions of the stimuli remained as consistent as possible.

Accuracy on the number line trials were measured using the percentage of absolute error formula (PAE = (|estimate - target|/scale of estimates) × 100%) as is generally used to evaluate number line estimation tasks.

### Mathematics anxiety

The Abbreviated Mathematics Anxiety Scale (AMAS) was used to measure mathematics anxiety (Hopko et al., 2003). This scale consists of nine self-report items which explore emotional responses to proposed mathematical tasks and scenarios. Answers are recorded on a 5 point Likert scale whereby 1 denotes ‘Low Anxiety’ and 5 denotes ‘High Anxiety’. Total scores are calculated using the sum of responses and can range from 9 to 45, whereby a higher score indicates higher anxiety. Reliability analysis in the current sample yielded a Cronbach’s Alpha of 0.902.

### Spatial anxiety questionnaire

The Spatial Anxiety Scale (SAQ) was used to gauge spatial anxiety (Lyons et al., 2018). This scale consisgts of eight self-report items examining emotional responses to situations involving spatial navigation or wayfinding. Responses are recorded on a 5 point Likert scale ranging from A “Not at all Anxious” to E “Very Anxious”. Total scores are calculated using the sum of responses which range from 8 to 40, whereby higher scores indicate higher levels of spatial anxiety. Reliability analysis in the current sample yielded a Cronbach’s Alpha of 0.881.

### Anxiety controls

The Generalised Anxiety Disorder Assessment (GAD7), the State-Trait Anxiety Inventory (STAI) short form and the Test Anxiety Inventory (TAI5) were used as covariates in the analysis with which to compare the outcomes of the mathematics and spatial anxiety scales. Information and instruction for the anxiety controls are covered in Supplementary Materials (Spitzer et al., 2006; Stöber & Pekrun, 2004; Zsido et al., 2020).

## Procedure

After reading the information sheet and responding to the consent form, participants first provided demographic information. They were next presented with the cognitive task instructions and a set of ten practice trials in which feedback was incorporated. Since a within-subjects design was adopted for this task, all participants completed all trials. Participants were instructed to complete the task as quickly as possible. A self-timed break of approximately 2 min was provided half way through the task following 48 trials. The full task took approximately 15 min to complete. Participants were requested to guess rather than leave a blank space, even in cases where they judged a calculation as difficult. Following completion of this task, participants completed the anxiety measures presented in random order. Finally, a debrief screen containing links for anxiety resources and contact details for the researcher was displayed.

## Results

### Data preparation

All statistical analyses were performed using IBM SPSS Statistics 24 and graphics using JASP, version 0.16 (JASP team, 2021). Initial data cleaning was first carried out to check participant engagement in the cognitive task. Of the initial 200 completed responses gathered on Gorilla between 31 May and 30 June 2021, 5 participants skipped > 30% of the trials, 2 participants provided inattentive responses to the trials, leaving overall accuracy and reaction times more than 3 standard deviations outside the mean. Seven cases were excluded, leaving 193 wholly completed responses within 3 standard deviations of the mean responses for accuracy and RT. A further 2 participants identified as non-binary on the demographic questionnaire, and 4 indicated that they preferred not to disclose their gender, therefore these participants were excluded from the gender analysis leaving 187. Finally, 3 participants completed the cognitive task only, meaning 184 participants were included in the exploration of the hypotheses relating to anxiety and gender. Of the remaining participants who fully completed the task, a total of 1.48% of trials were left blank.

### Initial exploration of the data

A full breakdown of participant demographics are presented in Table 1. Descriptive statistics for both the cognitive task and anxiety measures are available in Supplementary Materials. STEM background participants comprised close to 40% of the sample. Despite finding no difference between STEM and non-STEM participants in terms of SA, F (6,183) = 0.529; as expected, STEM participants did display significantly lower MA scores than their non STEM counterparts, F (6,183) = 4.36, *p* =.029. Further exploratory analyses relating to STEM background are reported in the Supplementary Materials.

In terms of the cognitive task, as expected, accuracy was high for the calculation component with a mean of 0.9, while accuracy on the number line trials yielded more variance with a mean error of 19.5%. Participants displayed the fastest reaction times for addition trials as expected with a mean of 7.1 s and most slowly for both division and subtraction trials with a mean of 7.7 s for both. Task performance is explored in more depth in the following sections. Table 2 presents correlations of all variables involved.

**Table 1:** Exp 1: participant demographic characteristics

Sample Characteristics	Female	Male	Overall
N(%)	119 (63.3)	68 (32.2)	187 (95.5)
Age, m(SD)	29.2 (10.32)	30.22 (10.88)	29.47(10.52)
Right – handed, n(%)	101 (84.9)	61 (88.4)	176 (90.3)
STEM background, n(%)	43 (36.1)	31 (44.9)	76 (39.0)

**Table 2:** Exp 1: Pearson’s correlation table of demographic and task variables

Measure	Math Anxiety	Spatial Anxiety	Calculation Control RT	Number line RT	Calculation Control Error
Spatial Anxiety	0.431**				
Calculation RT	0.294**	0.098			
Number Line RT	0.150*	0.032	0.500**		
Calculation Control Error	− 0.099	− 0.073	− 0.065	0.147*	
Number Line Error	− 0.206**	− 0.117	− 0.056	0.043	0.418**

**Correlation is significant at the 0.01 level (2-tailed)

*Correlation is significant at the 0.05 level (2-tailed)

### Directional performance statistics

Two initial repeated measures ANOVAs were implemented to determine differences in error and reaction time (RT) on the number line estimation task implemented in Experiment 1.

Using percentage absolute error (PAE) for each of the four directions left-right (LR); right-left (RL); bottom-top (BT) and top-bottom (TB) as a repeated measures variables, a significant difference was found for directional error, F(3,179) = 25.776, *p* =.001.

Post hoc tests with Bonferroni correction found that participant error was significantly lower on the LR condition than for any other condition, RL, t = −4.250, *p* =.001,d =−0.37, 95% CI [−0.61, −0.13]; BT, t = −3.10, *p* =.013,d =−0.27, 95% CI [−0.24, 0.18] or TB, t = − 8.73, *p* =.001,d = − 0.74, 95% CI [−0.99, 0.49].

BT error was also significantly lower than the TB condition, t = −5.39, *p* =.001, d = −0.47, 95% CI [−0.71, −0.23], but not RL, t = 1.25, *p* = 1.00, d = 0.10, 95% CI [−0.12, 0.32].

In summary, participant error was lower on the LR direction than for any other direction, while BT error was also significantly lower than for TB direction. Error was comparably high for both RL and TB conditions.

A similar repeated measures ANOVA was conducted to examine differences in reaction time (RT) across directions again finding a significant difference between conditions, F(3,179) = 4.764 *p* =.003.

Post hoc tests with Bonferroni correction confirmed that participant RT was significantly quicker on the BT condition than for TB, t = 4.056, *p* =.001,d = 0.18, 95% CI [0.06, 0.29], although no other condition differed significantly from the other for RT.

In summary, participants were quicker overall on the BT condition than for any other direction in this instance, though this difference only reached significance with the TB condition.

### Hypothesis 1 - mathematics anxiety and spatial anxiety on task reaction time and accuracy

We assessed the effects of MA and SA on both the number line trials and calculation controls. To do this, one multivariate multiple regression model was conducted, allowing us to examine both error and reaction time data for both number line and calculation control task components as they relate to each other. AMAS and SAQ total scores acted as predictor variables and calculation control RT, number line RT, calculation control accuracy and number line accuracy as the four dependent variables. Anxiety controls (GAD, TAI, STAIS, STAITS) were included as covariates in the model. An examination of the collinearity statistics for the predictor variables reported tolerance at 0.814 and a variance inflation factor of 1.228, indicating that multicollinearity would not be an issue of concern between variables. For clarity, the results for reaction time at documented first for each of the variables, followed by results for accuracy.

#### Reaction time

AMAS scores were found to be significant predictors of reaction time for calculation trials, *R²* = 0.087, *F*(6, 183) = 10.81, *p* =.001. Parameter estimates indicate that higher anxiety was related to slower RT, *β* = 68.25, *SE* = 20.71, *t* = 3.30. Conversely, SAQ scores did not exert a notable effect on reaction time for the calculation trials, *F*(6, 183) = 0.34, *p* =.559. TAI was the only anxiety control that significantly related to calculation RT in the model, *F*(6, 183) = 4.89, *p* =.001. AMAS scores were not significantly related to RT for number line trial components, *R²* = 0.019, *F*(6, 183) = 1.15, *p* =.285. Again, SAQ scores were not significantly related to number line trials, *F*(6, 183) = 0.87, *p* =.353. TAI scores were the only covariate measure to be significant for number line RT, *F*(6, 183) = 1.96, *p* =.014, where higher TAI was associated with higher RT, *β* = 87.13, *SE* = 33.85, *t* = 2.58.

#### Accuracy

The multivariate regression model found AMAS scores to be significant predictors of number line error, *R²* = 0.045, *F*(6, 183) = 5.12, *p* =.025, with higher reported MA relating to higher number line error, *β* = 3.08, *SE* = 1.51, *t* = 5.30. No significant effect was found for spatial anxiety on number line accuracy, *F*(6, 183) = 0.02, *p* =.903. The relationship between AMAS and calculation error was not significant, *R²* = 0.016, *F*(6, 183) = 1.63, *p* =.204, nor was the relationship between SAQ and calculation error, *F*(6, 183) = 0.23, *p* =.633. None of the included anxiety control measures significantly predicted accuracy for either calculation or number line trials Table 3.

**Table 3:** Exp 1: Synthesised regression results for Hypothesis 1

Dependent Variable	Anxiety Scale	*R*²	F(df)	Significant Covariates
Calculation RT	AMAS	0.087	F(2,183) = 10.81***	TAI (*p* =.001)
Calculation RT	SAQ	0.087	F(2,183) = 0.34	
Number Line RT	AMAS	0.019	F(2,183) = 1.15	TAI (*p* =.014)
Number Line RT	SAQ	0.019	F(2,183) = 0.87	
Calculation Error	AMAS	0.016	F(2,183) = 1.63	
Calculation Error	SAQ	0.016	F(2,183) = 0.23	
Number Line Error	AMAS	0.045	F(2,183) = 5.12*	
Number Line Error	SAQ	0.045	F(2,183) = 0.02	

**Correlation is significant at the 0.01 level (2-tailed)

*Correlation is significant at the 0.05 level (2-tailed)

To further investigate potential orientation-specific effects, we conducted exploratory regressions predicting directional number line performance from math and spatial anxiety. These analyses are intended to be indicative of trends in the data only and should be interpreted with caution, since the power analysis was conducted to estimate statistical power for our sample with the task in its entirety. AMAS scores were found to significantly predict error in the left-to-right, *F*(1, 138) = 9.42, *p* =.003, and bottom-to-top conditions, *F*(1, 138) = 5.52, *p* =.020. The effect of AMAS on right-to-left error approached significance, *F*(1, 138) = 3.71, *p* =.056. No significant associations emerged between SA and error. Neither MA nor SA significantly predicted directional reaction time. A full breakdown of results across all directional conditions is provided in Supplementary Materials.

### Exploratory analysis - gender difference in task performance, MA and SA

An initial exploratory examination of differences in anxiety scores alone showed that women reported significantly higher levels of anxiety on both the AMAS, *R²* = 0.028, *F*(1, 183) = 5.30, *p* =.023, and SAQ, *R²* = 0.042, *F*(1, 183) = 7.90, *p* =.005, scales than men. In spite of this, when gender was included in the multivariate regression model used to assess Hypothesis 1, results indicated no significant difference between men and women at the *p* <.05 level in either RT for calculation, *R²* = 0.090, *F*(2, 183) = 0.03, *p* =.874, or number line trials, *R²* = 0.025, *F*(2, 183) = 0.10, *p* =.753. Similar results were obtained for error in either calculation, *R²* = 0.014, *F*(2, 183) = 0.51, *p* =.822, or number line trials, *R²* = 0.054, *F*(2, 183) = 1.07, *p* =.303. These results suggest that, despite gender differences in anxiety levels, neither MA nor SA contributed to gender differences in any aspect of task performance Table 4.

**Table 4:** Exp 1: Synthesised regression results for exploratory gender analysis

Dependent Variable	*R*²	F(df)
AMAS	0.028	F(1,183) = 5.30*
SAQ	0.042	F(1,183) = 7.90*
Calculation RT	0.09	F(2,183) = 0.03
Number Line RT	0.025	F(2,183) = 0.10
Calculation Error	0.014	F(2,183) = 0.05
Number Line Error	0.054	F(2,183) = 1.07

## Discussion

Analysis of the data concluded that while MA was a determinant of both arithmetic and spatial elements of task performance (calculation controls and directional number line trials), no relationship was found between SA and any of the task components. One possibility is that this discrepancy between MA and SA’s association with directional number line estimation is because the concept of a 0-100 number line may be automatised in some adults. One point to note is the relatively high rate of error for number line estimation observed in this experiment. Although the 0–100 number line is a familiar numerical range for adults. Given the familiarity of this range, participants may have relied on quick or automatised estimation approaches, which proved less reliable when spatial frames of reference were manipulated. Conversely, participants may have perceived the 0–100 task as straightforward and engaged less analytically than on a more challenging or novel task.

Secondly, the parameters of this task may lack complexity required to tap into the processes of spatial processing or magnitude judgement associated with spatial anxiety, even when accounting for the directional element. It remains to be seen how an emphasis on numerical and spatial complexity may impact the relationship of both MA and SA with number line performance.

## Experiment 2

### Methods

#### Participants

Participants for this experiment were recruited in the same manner as for *Experiment 1* above. The participants were 150 adults recruited online (Mean age = 31.5, SD = 12.32, 51.1% women). The percentage of participants who reported having a career or expertise in STEM was 24.8%, while 54% of participants reported having obtained third level education. No participants reported a diagnosis of dyscalculia or a mathematics learning disorder.

### Materials and measures

**Fig. 2:**
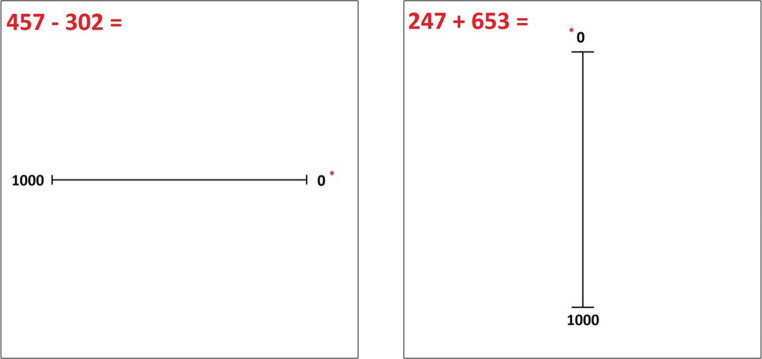
Example trials for the 0-1000 number line

The design, measures and procedures for this Experiment remained largely similar to those implemented in *Experiment 1*. The only changes are in the range of the number line in each trial, as well as the range of the calculation controls. In light of the results of the previous experiment, we have also lessened the number of anxiety controls whereby only TAI and GAD are included as covariates in the following analysis. In this experiment, the range is extended to 0-1000, meaning when controlling for numerical ability, participants answer calculations with responses ranging from 0 to 1000, and in each of the 48 number line trials, participants must place a target number between 0 and 1000 on a number line of the same length and parameters as that in Experiment 1 as seen in Fig. 2.

## Results

### Data Preparation

Of the 150 completed responses gathered on Gorilla between 31 May and 30 June 2021, 2 participants skipped > 30% of the trials. 4 further participants failed to respond to the anxiety questionnaires. Six cases were excluded, leaving 144 wholly completed responses within 3 standard deviations of the mean responses for accuracy and RT. A further 2 participants identified as non-binary on the demographic questionnaire, and 1 indicated that they preferred not to disclose their gender, therefore these participants were excluded from the gender analysis leaving 141 for analysis. STEM background was predictive of MA with non-STEM participants reporting higher levels of MA, *F*(1,135) = 4.31, *p* =.04, while SA again was not related to STEM background in this experiment *F*(1,135) = 3.08, *p* =.08. Further exploratory analyses relating to STEM background can be found in Supplementary Materials.

A full breakdown of participant demographics are presented in Table 5. Descriptive statistics for both the cognitive task and anxiety measures are available in Supplementary Materials. Accuracy on number line trials was slightly higher than the first experiment with a mean error of 10.7%, while mean accuracy on calculation controls was unsurprisingly slightly lower at 87.3%. Table 6 presents correlations of all variables involved.

**Table 5:** Exp 2: Participant demographic characteristics

Sample Characteristics	Female	Male	Overall
N(%)	70 (49.6)	65 (46.1)	141
Age, m(SD)	38.3 (12.96)	31.72 (13.20)	35.01(13.52)
Right – handed, n (%)	61 (87.1)	54 (83.1)	117 (83.0)
STEM background, n(%)	9 (12.9)	24 (36.9)	34 (24.8)

**Table 6:** Exp 2: Pearson’s Correlation table of demographic and task variables

Measure	Math Anxiety	Spatial Anxiety	Calculation Control RT	Number Line RT	Calculation Control Error
Spatial Anxiety	0.453**				
Calculation Control RT	0.295**	0.098			
Number Line RT	0.0001	− 0.126	0.530**		
Calculation Control Error	− 0.258**	− 0.142	− 0.146	0.258**	
Number Line Error	0.263**	0.179*	0.147	− 0.168*	− 0.393**

**Correlation is significant at the 0.01 level (2-tailed)

*Correlation is significant at the 0.05 level (2-tailed)

### Directional performance statistics

Two initial repeated measures ANOVAs were implemented to determine differences in error and reaction time (RT) on the number line estimation task implemented in Experiment 2.

Using percentage absolute error (PAE) for each of the four directions (LR, RL, BT and TB) as a repeated measures variables, a significant difference was found for directional error, F(3,140) = 5.010, *p* =.002.

Post hoc tests with Bonferroni correction found that participants error was significantly lower on the LR condition than for RL, t = −3.330, *p* =.007,d =−0.35, 95% CI [−0.63, −0.06] However, LR did not significantly differ from error on the BT condition, t = −0.351, *p* = 1.00,d =−0.04, 95% CI [−0.24, 0.18] or the TB condition, t = − 1.347, *p* = 1.00,d = − 0.12, 95% CI [−0.34, 0.12].

BT error was also significantly than the RL condition, t = 3.514, *p* =.004, d = 0.32, 95% CI [0.07, 0.57], but not TB, t = 0.850, *p* = 1.00, d = 0.092, 95% CI [−0.20, 0.38]. Similarly, RL and TB did not differ from each other, t = 1.871, *p* =.38, d = 0.23, 95% CI [−0.1, 0.56].

In summary, participant error was lower on the LR direction than for any other direction, while BT error was also significantly lower than for RL direction. Error was comparably high for both RL and TB conditions.

A similar repeated measures ANOVA was conducted to examine differences in reaction time (RT) across directions again finding a significant difference between conditions, F(3,140) = 3.157, *p* =.02.

Post hoc tests with Bonferroni correction confirmed that participant RT was significantly lower on the LR condition than for TB, t = −2.74, *p* =.03,d =−0.12, 95% CI [−0.22, −0.04], although no other condition differed significantly from the other for RT.

In summary, participants were significantly quicker from the LR condition than for the ‘incongruent’ vertical condition, TB. However, they were comparably quick across all other conditions in this instance.

#### Hypothesis 2 - mathematics anxiety and spatial anxiety on task reaction time and accuracy

We assessed the effects of MA and SA on number line performance. Similar to the previous experiment, a single multivariate regression model was implemented using AMAS and SAQ total scores as predictor variables and calculation control trial RT, number line RT, calculation control trial error and number line error as the four dependent variables in the model. Anxiety controls (TAI and GAD) were included as a covariate in each model.

##### Reaction time

Understandably, AMAS scores were found to be significant predictors of RT for calculation controls, *R²* = 0.13, *F*(4, 136) = 11.53, *p* =.001, with parameter estimates indicating that higher reported MA was related to slower RTs, β = 1818.71, *SE* = 916.65, *t* = 1.98. SAQ scores were not significantly related to RT for calculations, *F*(4, 136) = 0.40, *p* =.527. AMAS scores were also significantly related to RT for number line trial components, *R²* = 0.026, *F*(4, 136) = 27.57, *p* =.001, where again, parameter estimates indicated that higher MA predicted higher RTs, β = 78.13, *SE* = 32.36, *t* = 2.24. SAQ was not significantly related to RT for number line trials, *F*(4, 136) = 2.64, *p* =.107, and none of the SAQ subsets were significantly related to number line RT either. Neither of the anxiety controls significantly predicted number line RT.

##### Accuracy

AMAS scores were significant predictors of calculation control error, *R²* = 0.091, *F*(4, 136) = 7.76, *p* =.006, with higher AMAS scores associated with greater overall error, β = 4.52, *SE* = 2.05, *t* = 5.91. SAQ total scores were not significantly associated with number line error, *F*(4, 136) = 0.21, *p* =.649. AMAS scores did significantly predict number line error, *R²* = 0.088, *F*(4, 136) = 5.23, *p* =.042, with higher MA corresponding to greater error, β = 2.35, *SE* = 1.15, *t* = 2.05. No effect of SAQ total score was found for calculation error, *F*(4, 136) = 0.04, *p* =.834. However, when examining SAQ subsets individually, a significant effect of the mental manipulation subset on number line error was observed, *R²* = 0.040, *F*(4, 136) = 5.45, *p* =.021. None of the included anxiety control measures significantly predicted accuracy on either the calculation or number line trials. See Table 7 for summarised results.

**Table 7:** Exp 2: Synthesised regression results for Hypothesis 2

Dependent Variable	Anxiety Scale	*R*²	F(df)
Calculation RT	AMAS	0.13	F(1,137) = 11.53***
Calculation RT	SAQ	0.13	F(1,137) = 0.40
Number Line RT	AMAS	0.026	F(1,137) = 27.57***
Number Line RT	SAQ	0.026	F(1,137) = 2.64
Calculation Error	AMAS	0.091	F(1,137) = 7.76*
Calculation Error	SAQ	0.091	F(1,137) = 0.04
Number Line Error	AMAS	0.088	F(1,137) = 5.23*
Number Line Error	SAQ	0.088	F(1,137) = 0.21

***Correlation is significant at the 0.01 level (2-tailed)

*Correlation is significant at the 0.05 level (2-tailed)

To explore whether directional number line performance was differentially associated with MA or SA in this experiment, we conducted regression analyses using the four directional error and RT measures. Results indicated that AMAS significantly predicted error in the left-to-right, *F*(1, 138) = 9.42, *p* =.003, and bottom-to-top conditions, *F*(1, 138) = 5.52, *p* =.020. The association with right-to-left error approached significance, *F*(1, 138) = 3.71, *p* =.056. No significant associations were observed between spatial anxiety and directional error. For reaction time, neither math nor spatial anxiety significantly predicted outcomes in any directional orientation. Again, these findings are indicative of trends in task performance only and should be interpreted cautiously. A complete table of results for all directional conditions is available in Supplementary Materials.

#### Exploratory analysis - gender difference in task performance, MA and SA

Again, female participants reported significantly higher levels of anxiety on both the AMAS, *R²* = 0.064, *F*(1, 137) = 3.51, *p* =.042, and SAQ, *R²* = 0.079, *F*(1, 137) = 4.80, *p* =.012, scales than their male counterparts. Yet, adding gender to the multivariate regression model in Hypothesis 2 suggests no significant difference between men and women at the *p* <.05 level in either RT for number line trials, *R²* = 0.026, *F*(1, 137) = 0.17, *p* =.914, or the calculation controls, *R²* = 0.13, *F*(1, 137) = 0.76, *p* =.519. The same pattern was observed for accuracy in both number line trials, *R²* = 0.088, *F*(1, 137) = 0.85, *p* =.471, and calculation controls, *R²* = 0.091, *F*(1, 137) = 1.02, *p* =.387. Table 8 below summarises results.

**Table 8:** Exp 1:Synthesised regression results for exploratory gender analysis

Dependent Variable	*R*²	F(df)
AMAS	0.064	F(1,137) = 3.51*
SAQ	0.079	F(1,137) = 4.80*
Calculation RT	0.13	F(1,137) = 0.76
Number Line RT	0.026	F(1,137) = 0.17
Calculation Error	0.091	F(1,137) = 1.02
Number Line Error	0.088	F(1,137) = 0.85

**Correlation is significant at the 0.01 level (2-tailed)

*Correlation is significant at the 0.05 level (2-tailed)

## Discussion

Having increased the numerical range in this adaptation of the task, it was initially postulated that the perceived complexity of the number line trials may also increase. Here, MA was indeed found to be predictive of both task accuracy and RT, which aligns with the expectation that higher task demand might trigger stronger effects of anxiety. However, in the current experiment, an increase in accuracy on number line trials was observed relative to Experiment 1. Although seemingly more spatially intricate due to the placement of three-digit numbers along a line of the same objective length as the 0-100 task, the proportional structure of a 0- 1000 number line remains similar to that of a 0 −100 line, and it is possible that this format remains relatively automated for most adults, particularly those with higher levels of education. Complexity in number line tasks may not be solely determined by numerical range, but more effectively manipulated by incorporating non-whole numbers, such as fractions or decimals, which may in turn place greater demands on spatial-numerical processing. Another possible explanation is that the larger numerical range encouraged participants to adopt more careful, proportional estimation strategies. In contrast to the potentially over-familiar 0–100 task, this condition may have elicited greater attentional engagement, thus leading to more accurate spatial placements.

Similar to the previous experiment, we see no significant impact of any facet of SA on number line performance, despite the outwardly spatial nature of the task. In this instance, we may have expected to see an impact of the Mental Manipulation or Imagery subsets of the SAQ, which measure aspects of small-scale spatial processing that relate to both numerical estimation and spatial rotation. Following Experiment 3, these findings will be reviewed collectively in the General Discussion. Furthermore, none of the anxiety controls exerted a significant impact on task performance here, which may support the interpretation of MA as a distinct emotional response elicited by mathematical tasks, even when spatial elements are prominent.

Here for a second time, we see a difference in the levels of both MA and SA between women and men, with women scoring higher on both anxiety measures, yet we see no discernible difference in cognitive task performance. It remains to be seen how a more drastic variation in the paradigm, switching to an unbounded number line, might tap into more refined aspects of spatial processing than the previous two designs and associate with both math anxiety and spatial anxiety.

### Experiment 3

#### Methods

##### Participants

Participants for this experiment were recruited in the same manner as for Experiments 1 and 2. Participants were 150 adults recruited online via Prolific and online advertisement (Mean age = 36.1, SD = 13.49, 50% women). 30.7% of participants reported having a career or expertise in Science Technology Engineering and Math (STEM) and 62.9% participants reported having completed third level education. No participants reported a diagnosis of dyscalculia or a mathematics learning disorder.

### Materials and measures

**Fig. 3:**
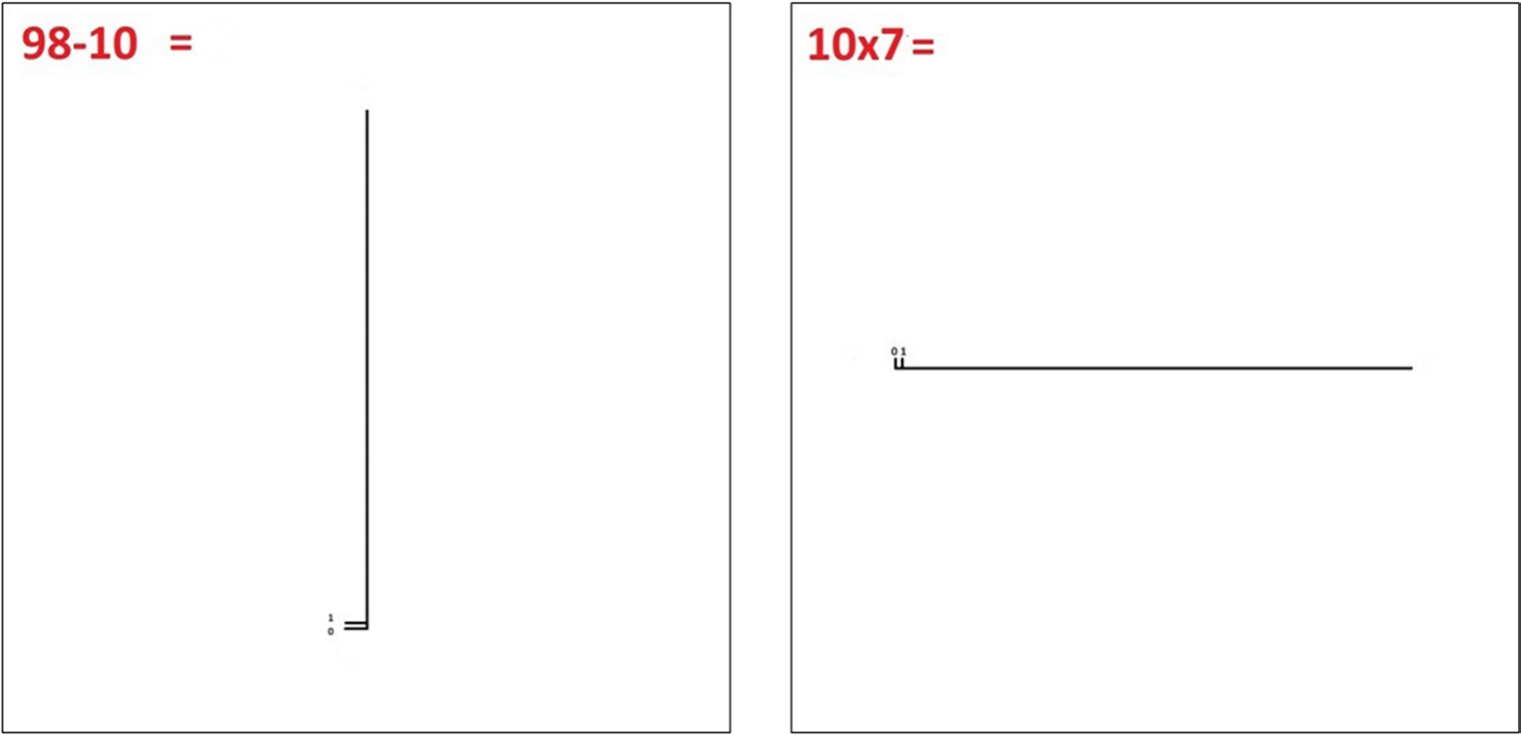
Example trial for the unbounded number line

The design, measures and procedures for this Experiment remained largely similar to those implemented in *Experiment 2*. The only change in this experiment is that participants are presented with an unbounded number line in each trial rather than a bounded number line. In this experiment, the range remains to 0-100 for calculation controls and number line targets. In each of the 48 number line trials, participants must place a target number on a number line of the same length as that in *Experiment 1*, but where endpoint is not indicated, only one unit of distance is indicated at the start point, as seen in Fig. 3.

## Results

### Data preparation

Of the 150 completed responses gathered, six participants skipped > 30% of the trials, two participants provided systematic responses to the trials, leaving overall accuracy and reaction times more than 3 standard deviations outside the mean. Two further participants failed to respond to the anxiety questionnaires. Ten cases were excluded in total, leaving 140 wholly completed responses within 3 standard deviations of the mean responses for accuracy and RT. Of this 140, 4 participants did not wish to disclose their gender, leaving 136 participants for the exploratory gender analysis. A full breakdown of participant demographics are presented in Table 9. Descriptives for both the cognitive task and anxiety measures are available in Supplementary Materials.

Average error on number line trials was comparable to the previous study with 10.9%. Performance on calculation control trials were comparable to the previous two experiments with an average accuracy of 92.0%. Table 10 presents correlations of all variables involved. STEM background was not significantly related to levels of MA in this experiment *F*(1,134) = 3.17, *p* =.08. However, a relationship between SA and STEM background was observed in this experiment, with non-STEM background being related to higher levels of SA, *F*(1,134) = 5.44, *p* =.021. Further analyses relating to STEM background for this experiment are documented in Supplementary Materials.

**Table 9:** Exp 3: Participant demographic characteristics

Sample Characteristics	Female Male Overall
N(%)	70 (50.0) 66 (47.1) 140
Age, m(SD)	33.4 (14.69) 31.72 (13.20) 40.73 (12.06)
Right – handed, n (%)	64(91.4) 56 (84.8) 120 (85.7)
STEM background, n(%)	13(18.6) 30 (45.5) 43 (31.6)

**Table 10:** Exp 3: Pearson’s Correlation table of demographic and task variables

Measure	Math Anxiety	Spatial Anxiety	Calculation Control RT	Number Line RT	Calculation Control Error
Spatial Anxiety	0.564**				
Calculation Control RT	0.262**	0.153			
Number Line RT	− 0.190*	− 0.076	0.409**		
Calculation Control Error	− 0.206*	− 0.097	− 0.071	0.192*	
Number Line Error	0.344**	0.163	0.008	− 0.255**	− 0.342**

**Correlation is significant at the 0.01 level (2-tailed)

*Correlation is significant at the 0.05 level (2-tailed)

#### Directional performance statistics

Two initial repeated measures ANOVAs were implemented to determine differences in error and reaction time (RT) on the number line estimation task implemented in Experiment 3.

Using percentage absolute error (PAE) for each of the four directions (LR, RL, BT and TB) as a repeated measures variables, a significant difference was found for directional error, F(3,139) = 10.97, *p* =.001.

Post hoc tests with Bonferroni correction found that participants error was significantly lower on the LR condition than for RL, t = −5.115, *p* =.001, d =−0.25, 95% CI [−0.40, −0.11]; and TB,, t = − 3.019, *p* =.018,d = − 0.15, 95% CI [−0.29, −0.02]. However, LR did not significantly differ from error on the BT condition, t = −1.252, *p* = 1.00,d =−0.05, 95% CI [−0.15, 0.06].

BT error also differed significantly from RL, t = 3.636, *p* =.002, d = 0.199, 95% CI [0.05, 0.39], but not TB, t = 2.039, *p* =.26, d = 0.10, 95% CI [−0.33, 0.24]. Similarly, RL and TB did not differ from each other, t = 2.51, *p* =.26, d = 0.10, 95% CI [−0.01, 0.26].

In summary, participant error was lowest on the LR condition, though error was comparably low on the BT condition, while error was higher overall on the RL and TB conditions.

A similar repeated measures ANOVA was conducted to examine differences in reaction time (RT) across directions again yielding a significant difference between conditions, F(3,139) = 8.80, *p* =.001.

Post hoc tests with Bonferroni correction found that participant RT was significantly lower on the LR condition than for all other conditions, RL, t = −3.33, *p* =.007,d =−0.11, 95% CI [−0.21, −0.02]; and TB,, t = − 4.31, *p* =.001,d = − 0.17, 95% CI [−0.27, −0.06], and the BT condition, t = −4.45, *p* =.001,d =−0.16, 95% CI [−0.27, −0.06]. No other condition differed significantly from the other for RT.

In summary, participants were significantly quicker from the LR condition than for the reversed condition or either vertical condition for the unbounded number line iteration of the task.

## Hypothesis 3 - mathematics anxiety and Spatial anxiety on task reaction time and accuracy

A single multivariate regression model was implemented using AMAS and SAQ total scores as predictor variables and number line and calculation RT and number line and calculation accuracy as dependent variables. Anxiety controls (GAD, TAI) were included as covariates in each model.

### Reaction time

In this experiment, AMAS scores were found to be significantly related to RT for calculation controls, *R²* = 0.071, *F*(4, 135) = 6.64, *p* =.011, where higher AMAS scores were associated with slower RTs, β = 49.61, *SE* = 23.23, *t* = 2.14. Though AMAS scores were not indicative of RT for number line trials, *R²* = 0.040, *F*(4, 135) = 1.31, *p* =.060, SAQ scores were not significantly related to RT for number line trials, *F*(4, 135) = 0.14, *p* =.710, or for calculation controls, *F*(4, 135) = 0.001, *p* =.985. In this instance, none of the SAQ subsets were significantly related to RT. None of the anxiety controls exhibited significant associations with RT for number line trials.

### Accuracy

AMAS scores were found to be significant predictors of both calculation error, *R²* = 0.061, *F*(4, 135) = 5.20, *p* =.024, where higher reported MA was indicative of higher overall error, β = 0.03, *SE* = 0.01, *t* = 2.20. Higher AMAS scores were also indicative of higher number line error in this experiment, *R²* = 0.123, *F*(4, 135) = 13.77, *p* =.001, β = 0.42, *SE* = 0.20, *t* = 3.81. SAQ scores did not significantly affect calculation error, *F*(4, 135) = 0.29, *p* =.589, or number line error, *F*(4, 135) = 0.34, *p* =.562. When individual subsets were examined, the SAQ Imagery component significantly affected number line accuracy, *R²* = 0.032, *F*(1, 139) = 4.57, *p* =.017. None of the included anxiety control measures were significantly related to prediction of accuracy for either calculation or number line trials. Results are summarised in Table 11.

**Table 11:** Exp 1: Synthesised regression results for Hypothesis 3

Dependent Variable	Anxiety Scale	*R*²	F(df)
Calculation RT	AMAS	0.071	F(1,139) = 6.64*
Calculation RT	SAQ	0.071	F(1,135) = 0.00
Number Line RT	AMAS	0.04	F(1,135) = 1.31*
Number Line RT	SAQ	0.04	F(1,135) = 0.14
Calculation Error	AMAS	0.061	F(1,135) = 5.20*
Calculation Error	SAQ	0.061	F(1,135) = 0.29
Number Line Error	AMAS	0.123	F(1,135) = 13.77***
Number Line Error	SAQ	0.123	F(1,135) = 0.34

**Correlation is significant at the 0.01 level (2-tailed)

*Correlation is significant at the 0.05 level (2-tailed)

Exploratory analyses which should be interpreted cautiously examining directional performance revealed that AMAS significantly predicted error in all four number line orientations for this experiment: top-to-bottom (*F*(1,137) = 14.42, *p* <.001), left-to-right (*F*(1,137) = 10.84, *p* =.001), right-to-left (*F*(1,137) = 13.49, *p* <.001), and bottom-to-top (*F*(1,137) = 12.16, *p* =.001). In terms of RT, AMAS was also significantly related in the left-to-right (*F*(1,137) = 7.05, *p* =.009) and top-to-bottom (*F*(1,137) = 4.00, *p* =.048) conditions. No significant effects of SAQ were observed on either error or RT. The full statistical results are available in Supplementary Materials*.*

## Exploratory Analysis - Gender difference in task performance, MA and SA

In this study, women’s reported math anxiety on the AMAS was not significantly higher than men’s, *R²* = 0.075, *F*(1, 135) = 0.61, *p* =.094. However, they did report significantly higher levels of spatial anxiety measured by the SAQ, *R²* = 0.075, *F*(1, 135) = 3.31, *p* =.024. For a third time, adding gender to the existing regression model indicated no significant differences between men and women in either RT for calculation, *R²* = 0.071, *F*(1, 135) = 0.12, *p* =.880, or number line trials, *R²* = 0.040, *F*(1, 135) = 0.13, *p* =.877, or in error for calculation, *R²* = 0.061, *F*(1, 135) = 1.29, *p* =.280, or number line trials, *R²* = 0.123, *F*(1, 135) = 0.27, *p* =.762. Results are summarised in Table 12.

**Table 12:** Exp 3: Synthesised regression results for exploratory gender analysis

Dependent Variable	*R*²	F(df)
AMAS	0.075	F(1,135) = 0.61
SAQ	0.075	F(1,135) = 3.31*
Calculation RT	0.071	F(1,135) = 0.12
Number Line RT	0.04	F(1,135) = 0.13
Calculation Error	0.061	F(1,135) = 1.29
Number Line Error	0.123	F(1,135) = 0.27

**Correlation is significant at the 0.01 level (2-tailed)

*Correlation is significant at the 0.05 level (2-tailed)

## Discussion

In this third experiment, we saw generally similar patterns to the previous two experiments in the study. In this adaptation, MA was significantly associated with number line accuracy on the unbounded number line in the directional paradigm, but was not significantly associated with reaction time. Overall, reaction time was a little higher on this task than for the previous two bounded versions in this study. Therefore, it may be the case that respondents were generally more careful in their completion of this task, the format of which would be largely more unfamiliar to most than a traditional number line, and most of their cognitive resources are allocated towards giving accurate responses. This in turn would cause feelings of MA to drive their reaction towards accuracy rather than timing. Number line error here was comparable with Experiment 2, suggesting that participants may have again relied on more proportional estimation strategies in the absence of explicit endpoints. This increased perceived novelty or complexity of the task may have promoted greater attentional engagement, contributing to lower error rates.

We do see a slight association between the Imagery subset of the Spatial Anxiety Questionnaire with number line accuracy in this experiment. This again may be due to the more unfamiliar nature of the unbounded number line, which when paired with a directional element may incorporate more visuo-spatial processing to complete than the bounded versions of the task. Still when examined as an overall indicator, spatial anxiety does not seem to relate to unbounded number line task performance. Furthermore, in this adaptation, none of the anxiety controls showed any association with task performance, furthering support for the premise of MA as a distinct construct from other forms of anxiety.

Finally, for the first time in this experiment, we observe no significant effect of gender on MA levels. MA is comparably high between both men and women here, yet still, women score significantly higher than men in SA with no significant difference in any aspect of task performance. This finding is discussed in detail along with our comprehensive results in the next section.

### General discussion

We developed three digital adaptations of a number line estimation task, all of which allowed us to switch the direction of the number line between trials and examined the extent to which both mathematics and spatial anxiety impacted the accuracy and reaction time of a sample of young adults, all of whom had learned mathematics through English. These three studies replicated findings already observed in children that digital number lines are a reliable measure with many ecological advantages concerning data storage, precision and real time scores. The present results in many ways contradict our hypotheses and in effect, previous propositions about the relation between numerical and spatial cognition.

Much previous literature supports the link between spatial and numerical processing, and the impact of MA is documented on many facets of mathematical learning (Beilock & Maloney, 2015; Georges et al., 2015; Maloney et al., 2012; Viarouge et al., 2014). In light of such evidence, it was expected that MA would impose an influence on both number line response time and accuracy (as well as calculation controls) in the current cognitive tasks. However, questions could be imputed regarding the lack of relationship determined between spatial anxiety and task elements. If cognitive facets of numerical and spatial thinking are deemed to be closely related, then why not also the measures of emotional components, in this case?

Despite examining performance on an outwardly spatial number line component of mathematical processing here, it did not seem to be affected by the participant’s reported SA in any variation of this task, yet all elements were affected by MA. Such results support recent findings for evidence of overlap between domain-specific anxieties, specifically MA and its significance in spatial performance (Ashkenazi & Cohen, 2021; . Ferguson et al., 2015). The results are suggestive of MA as an inhibitor which extends beyond that of other domain specific anxieties, although this cross-sectional paradigm was not designed specifically to test this. Indeed, it may be this negative emotive response to mathematics which predicts spatial-numerical performance more so than affect towards spatial-processing.

An additional explanation for the more consistent influence of MA over SA in the current study may relate to the structure and framing of the number line estimation task itself. Although the task required participants to map a value spatially, it followed immediately after an arithmetic calculation involving the same number. This design may have inadvertently activated more numerical rather than spatial strategies, with participants potentially carrying over their mental representation of the answer into the estimation trial. This sequencing could have made the task feel more mathematical in nature, thereby intensifying math-related anxiety while downplaying the relevance of spatial strategies or concerns. It may be possible that participants perceived the task overall as being more numerically demanding than spatial, even in trials requiring visuospatial placement. As anxiety is often driven by self-perceived difficulties, the framing around the perceived domain of the task may play a crucial role in determining which emotional response is triggered. If participants understood the estimation trials as mathematical problems rather than spatial judgments, this may help to explain the more prominent role of MA over SA in task performance. Future research may benefit from exploring how task framing or instructions modulate the involvement of domain-specific anxieties.

Furthermore, test anxiety was yielded as the only significant covariate in the prediction of MA on task reaction time, though this was only observed in *Experiment 1*. This finding might support research postulating a relationship between MA and test anxiety to a further extent than general or state anxiety. In other words, it is possible that MA relates to a fear of failure or “being exposed” and academic self-doubt beyond the more pathological aspects of anxiety. Test anxiety is often considered the form of anxiety most closely related to MA, with significant overlap often observed between the two constructs, raising the argument of how separate these constructs really are (e.g. Devine et al., 2012). Yet, although MA was a predictor of number line accuracy in the above studies, test anxiety did not play a significant role in accuracy for any of the experiments above. This could suggest that MA may be working as a separate construct on number line trials in the present study, calling for further investigation into the mechanisms of MA and its relationship to other types of anxiety in visuospatial aspects of mathematical processing.

It seems important to note further, that in all of the studies presented here, no effect of gender was found on any aspect of cognitive performance. Although average levels of both SA and MA were reported to be quite high across all participants in all three experiments (which is indeed a topic worthy of a separate discussion), women generally displayed significantly higher levels of both MA and SA than men. It is not hugely surprising to see a lack of gender difference in overall task performance. While it should be noted that many studies do find gender-specific differences in facets of mathematics performance; for example, boys have previously been found to outperform girls on tasks requiring knowledge of measurements, or restructuring or reorganisation of numbers (Innabi & Dodeen, 2018; Van den Heuvel-Panhuizen, 2004), other studies of numerical performance provide evidence on the contrary (e.g. Hyde et al., 1990; Kersey et al., 2018). For instance, the underestimation of high achieving girls by teachers and mothers which can otherwise deter women from pursuing careers or enterprise in STEM fields. (Devine et al., 2012; Hyde, 2016; Hutchinson et al., 2018; McCoy et al., 2021). Nevertheless, the current results are reminiscent of others in the literature in that time and time again, gender differences are apparent in domain specific emotional components of mathematics, putting women more strongly at risk for consequences on achievement and the associated after-effects (Foley et al., 2017). In fact, it is postulated that despite several previous observations of slim difference among genders in mathematics performance, women often report higher levels of MA due to self-doubt, discrepancies in academic encouragement and other implications of societal compartmentalization (Maloney et al., 2012; Stoet et al., 2016). This theory is bolstered by the current findings, in which women reported significantly higher levels of anxiety across both mathematics and spatial anxiety scales, which is especially curious considering the lack of interaction between spatial anxiety and task performance.

The present results found no gender difference in numerical-spatial performance, only in measure of emotional response to numerical and spatial elements of the task. Still, it is hypothesised that gender plays a large role in how spatial processes are framed and embodied, leading to discrepancies in performance depending on the task presentation (Tarampi et al., 2016). Perhaps such perceptual differences could be considered when exploring spatial-numerical associations and how the framing of such spatial- numerical processes might relate to reported anxiety which occurs in response to these tasks.

### Limitations and future research

One supposed constraint of the study was the fact it was administered online, which was necessary throughout the COVID-19 pandemic, but meant we were rather limited in controlling and observing the ways in which participants engaged with the task. That being said, results obtained from the three variations of this multi-directional number line task were in general, quite consistent as they related to both cognitive performance outcomes and in their relationships to domain-specific anxieties and gender which were the focus of this study. This might suggest that number line estimation tasks can be run in digital and online environments provided all variables are counterbalanced, the task is of a manageable duration and is accompanied with clear instructions and inclusion criteria as above. Still, it would certainly be beneficial to administer this study in lab, classroom and clinical settings. This might allow for an even more ecologically valid examination of the role of anxiety in spatial aspects of maths performance.

It is also important to consider differences in the structure and content of the domain-specific anxiety measures used. The AMAS includes subscales that assess both anxiety related to learning and being evaluated in mathematics, potentially capturing a broader range of emotional responses. In contrast, the SAQ used in this study primarily assesses anxiety directly arising from large and small scale spatial abilities and does not contain a specific subscale for performance or evaluation. This structural difference may partially explain the stronger predictive power of MA, particularly if participants experienced the number line task as evaluative in nature. Future studies could benefit from incorporating some form of spatial anxiety measure including an evaluation subscale to allow for balanced comparisons.

In addition, no initial task was administered to control for spatial ability and this process was only examined in the context of spatial-numerical processing. Secondly, to test the multiple experimental conditions in the cognitive task using a means that was applicable to as many populations as possible, the initial task design restricted the number line stimuli to 0-1000 in the more complex *Experiment 2* as a proof of concept. For instance, an even more abnormal number line could be used in this paradigm to examine the questions of complexity and range, since working with a 0-1000 number line in a sample of adults may not elicit symptoms of MA to the same extent that more complex estimations might, particularly in the current sample whereby many of the participants noted completing substantial levels of education. It is also possible that a 0-1000 number line might still be somewhat automatised for an adult participant, similar to the 0-100 condition discussed above, resulting in less effort on the spatial component. Subsequent research might aim to use a similar design across more complex numerical ranges which may result in more robust interactions of specific task conditions with presentations of MA (Georges et al., 2016; Starling-Alves et al., 2021). Alternatively, it may be of interest to administer the current task design in a sample of already extensively mathematically or spatially anxious individuals and compare responses with low anxiety samples. This may allow for a more controlled exploration of how specific conditions or elements within the task are impacted by anxiety. One further limitation of the current study is that information on socioeconomic status was not collected. Given, the established link between facets of numerical performance and socioeconomic background, this information would be useful to consider in future studies.

The findings presented here offer meaningful implications for both theory and practice. Theoretically, the study adds nuance to our understanding of how emotional factors, particularly MA, interact with spatial-numerical processing. The dissociation between SA and task performance suggests that emotional interference in mathematical contexts may indeed be domain-specific, with MA functioning as a broader inhibitor across both arithmetic and visuospatial domains. Practically, these insights may inform the design of educational tools and interventions aimed at reducing anxiety by incorporating spatial tasks in a way that does not exacerbate emotional stress. The current task’s digital format and adaptability may also serve as a valuable tool in classroom and remote learning settings to identify and support those experiencing elevated anxiety levels during mathematical processing.

With this in mind, potential avenues of future research might involve taking advantage of the nature of the numerical-spatial task designed for this study to advance insights on the role of spatial processing in holistic mathematical cognition and development. The controlled design of the task means that components can be isolated, developed or refined to explore specific research questions. For example, the multidirectional and aural elements may make it a useful tool in examining the strength of rigidity of the mental number line in different developmental, linguistic or even clinical populations. To explore this further, current studies are underway to investigate differences of language direction on directional number line performance in both Western and non-Western samples.

## Conclusions

The present study implemented an adapted multi-directional number line paradigm which allowed us to gain new insight into the associations between emotional components impacting both spatial and numerical aspects of mathematical processing. Our findings conclude that MA links to both arithmetic and visuospatial elements of mathematics performance, offering insight into the multi-faceted nature of emotional numerical processing and the elements which must be taken into consideration in attempts to alleviate MA. The additional examination of SA further complexifies the argument, where in this case, it did not relate to either aspect of task performance, prompting discussion surrounding the extent to which spatial and numerical processes may be linked through the role of individual difference in such processes. The multi-directional number line paradigm is implemented here to assess emotional components of spatial numerical associations and might be of use in further research on processes relating to the mental number line among different populations.

## Supplementary Information

Below is the link to the electronic supplementary material.

Supplementary Material 1

## Data Availability

A link to the data will be provided in the manuscript file.
