# Supplementary material for: Diverging roles of domain-specific anxieties in number-space associations. Insights from a multi-directional number line paradigm
Source: Psychol Res. 2025 Sep 30;89(5):149. doi: 10.1007/s00426-025-02179-0 (PMC12484368; doi:10.1007/s00426-025-02179-0)
Supplement: Supplementary file 1 — Supplementary Material 1 [file 426_2025_2179_MOESM1_ESM.docx]

**Supplementary Materials**

**Additional Hypotheses:**

Hypothesis 2A: Reaction times will be quicker when responding to left-right number line trials which are congruent with a left-to-right internal Mental Number Line in a Western, English speaking, sample.

Hypothesis 2B: Accuracy will also be higher when responding to left-right number line trials.

Hypothesis 3A: Reaction time will differ significantly between auditory and visual modality trials due to individual differences in modality processing.

Hypothesis 3B: Accuracy will also differ significantly between auditory and visual modality trials.

**Anxiety Controls:**

GAD7: The GAD7 acts as a self-report screening tool and severity measure for Generalised Anxiety Disorder (Spitzer et al., 2006). Participants rate on a 4-point Likert scale, how often they have been bothered by a series of 7 scenarios over the previous 2 weeks. Scores range from 0-21 with higher scores indicating more severe levels of generalised anxiety.

TAI5: The TAI5 is a short form of the Test Anxiety Inventory (Taylor and Deane, 2002). Participants rate on a 4-point Likert scale which assesses how frequently symptoms of anxiety are experienced before, during and after testing and examinations. Scores range from 5 to 20 with higher scores indicating more anxiety.

STAI-T/STAI-S: The short form State Trait Anxiety Inventory (Zsido et al., 2020; Spielberger et al., 1983) assesses symptoms of state and trait anxiety using a 4 point Likert. The scale consists of 10 items in total; 5 assessing state anxiety and 5 assessing trait anxiety. Scores range from 0 to 15 on each scale with higher scores indicating more severe anxiety symptoms.

| **Sample Characteristics** | ***Female*** | ***Male*** | ***Overall*** |
| --- | --- | --- | --- |
| AMAS, m(SD) | 25.55(8.42) | 21.56(8.56) | 24.46 (8.48) |
| SAS, m(SD) | 24.18(7.48) | 21.09(6.61) | 23.1 (7.34) |
| GAD, m(SD) | 15.46(6.14) | 15.54(6.03) | 8.56(6.03) |
| TAI, m(SD) | 13.4(4.48) | 11.84(4.18) | 12.92(4.41) |
| STAIS, m(SD) | 7.52(2.93) | 7.27(2.97) | 7.47 (2.93) |
| STAIT, m(SD) | 12.48(4.15) | 12.39(4.25) | 12.55 (4.17) |
| Task: Arithmetic  Accuracy, m(SD) | 0.91(0.96) | 0.90(0.95) | 0.90 (0.098) |
| Task: Arithmetic  RT, m(SD) | 7548.75(2128.67) | 7635.76(2393.11) | 7573.35 (2189.28) |
| Task: Number Line Accuracy, m(SD) | 0.56(0.14) | 0.54(0.17) | 0.55 (0.146) |
| Task: Number Line RT, m(SD) | 3607.8(1185.6) | 3651.76(1968.27) | 3623.32 (1496.49) |

**Performance data on multi-directional number line task**

**Experiment 1**

| Anxiety Scale | Performance Measure | Direction | *F* | *R²* |
| --- | --- | --- | --- | --- |
| AMAS | Error | LR | 9.42* | .067 |
|  |  | RL | 3.71* | .062 |
|  |  | TB | .58 | .004 |
|  |  | BT | 5.52* | .081 |
|  | RT | LR | 1.18 | .023 |
|  |  | RL | 0.37 | .022 |
|  |  | TB | 0.58 | .012 |
|  |  | BT | 0.28 | .020 |
| SAQ | Error | LR | 0.62 | .067 |
|  |  | RL | 1.47 | .062 |
|  |  | TB | 0.24 | .004 |
|  |  | BT | 1.52 | .081 |
|  | RT | LR | 3.17 | .023 |
|  |  | RL | 3.11 | .022 |
|  |  | TB | 1.58 | .012 |
|  |  | BT | 2.78 | .020 |

***S.1*** Multivariate regression results from *Experiment 1* showing relationship between AMAS and SAQ on directional number line estimation accuracy and reaction time (RT) across four spatial orientations, where *** denotes significance at the level p < 0.001, while * denotes significance at the level p < 0.05.

**Experiment 2**

| Anxiety Scale | Performance Measure | Direction | *F* | *R²* |
| --- | --- | --- | --- | --- |
| AMAS | Error | LR | 10.84*** | .067 |
|  |  | RL | 4.59* | .062 |
|  |  | TB | 0.30 | .004 |
|  |  | BT | 6.08* | .081 |
|  | RT | LR | 7.05 * | .060 |
|  |  | RL | 3.45 | .025 |
|  |  | TB | 4.00 * | .037 |
|  |  | BT | 3.15 | .027 |
| SAQ | Error | LR | 0.62 | .067 |
|  |  | RL | 1.47 | .062 |
|  |  | TB | 0.29 | .005 |
|  |  | BT | 1.52 | .081 |
|  | RT | LR | 3.17 | .023 |
|  |  | RL | 3.11 | .022 |
|  |  | TB | 1.58 | .012 |
|  |  | BT | 2.73 | .020 |

***S.2*** Multivariate regression results from *Experiment 2* showing relationship between AMAS and SAQ on directional number line estimation accuracy and reaction time (RT) across four spatial orientations, where *** denotes significance at the level p < 0.001, while * denotes significance at the level p < 0.05.

**Experiment 3**

| Anxiety Scale | Performance Measure | Direction | *F* | *R²* |
| --- | --- | --- | --- | --- |
| AMAS | Error | LR | 10.84*** | .098 |
|  |  | RL | 13.49*** | .112 |
|  |  | TB | 14.42*** | .127 |
|  |  | BT | 12.16*** | .091 |
|  | RT | LR | 7.05* | .060 |
|  |  | RL | 3.43 | .025 |
|  |  | TB | 4.00* | .037 |
|  |  | BT | 3.15 | .027 |
| SAQ | Error | LR | 0.03 | .098 |
|  |  | RL | 0.22 | .112 |
|  |  | TB | 0.05 | .127 |
|  |  | BT | 0.91 | .091 |
|  | RT | LR | 0.19 | .06 |
|  |  | RL | 0.65 | .025 |
|  |  | TB | 0.04 | .037 |
|  |  | BT | 0.13 | .027 |

***S.3*** Multivariate regression results from *Experiment 3* showing relationship between AMAS and SAQ on directional number line estimation accuracy and reaction time (RT) across four spatial orientations, where *** denotes significance at the level p < 0.001, while * denotes significance at the level p < 0.05.

**Exploration of STEM background and study variables**

|  | Exp 1 | Exp 2 | Exp 3 |
| --- | --- | --- | --- |
| Arithmetic Error | t (178) = 0.28 | t (135) = 0.08 | t (134) = 1.66 |
| Arithmetic RT | t (178) = -1.69 | t (135) = -0.15 | t (134) = -0.29 |
| Number Line Error | t (178) = 0.14 | t (135) = -1.6 | t (134) = -0.39 |
| Number Line RT | t (178) = 0.40 | t (135) = -0.21 | t (134) = -1.27 |
| Gender x STEM | χ²(1) = 0.49 | χ²(1) = 13.83* | χ²(1) = 11.355*** |

***S.4*** Exploratory analyses examining the relationship between STEM background and number line task performance, and gender across Experiments 1 – 3, where *** denotes significance at the level p < 0.001, while * denotes significance at the level p < 0.05.
